# LATP-Enhanced Polymer Electrolyte for an Integrated Solid-State Battery

**DOI:** 10.3390/polym17192673

**Published:** 2025-10-02

**Authors:** Xianzheng Liu, Nashrah Hani Jamadon, Liancheng Zheng, Rongji Tang, Xiangjun Ren

**Affiliations:** 1 College of Mechanical Engineering, Shandong Huayu University of Technology, Dezhou 253034, China; 2 Department of Mechanical and Manufacturing Engineering, Faculty of Engineering and Built Environment, Universiti Kebangsaan Malaysia, Bangi 43600, Malaysia; p159501@siswa.ukm.edu.my (L.Z.); p159500@siswa.ukm.edu.my (R.T.); p159404@siswa.ukm.edu.my (X.R.)

**Keywords:** polymer electrolytes, integrated electrode, conductive materials, solid electrolyte, lithium-ion battery

## Abstract

Traditional liquid electrolyte batteries face safety concerns such as leakage and flammability, while further optimization has reached a bottleneck. Solid electrolytes are therefore considered a promising solution. Here, a PEO–LiTFSI–LATP (PELT) composite electrolyte was developed by incorporating nanosized Li_1.3_Al_0.3_Ti_1.7_(PO_4_)_3_ fillers into a polyethylene oxide matrix, effectively reducing crystallinity, enhancing mechanical robustness, and providing additional Li^+^ transport channels. The PELT electrolyte exhibited an electrochemical stability window of 4.9 V, an ionic conductivity of 1.2 × 10^−4^ S·cm^−1^ at 60 °C, and a Li^+^ transference number (tLi+) of 0.46, supporting stable Li plating/stripping for over 600 h in symmetric batteries. More importantly, to address poor electrode–electrolyte contact in conventional layered cells, we proposed an integrated electrode–electrolyte architecture by in situ coating the PELT precursor directly onto LiFePO_4_ cathodes. This design minimized interfacial impedance, improved ion transport, and enhanced electrochemical stability. The integrated PELT/LFP battery retained 74% of its capacity after 200 cycles at 1 A·g^−1^ and showed superior rate capability compared with sandwich-type batteries. These results highlight that coupling LATP-enhanced polymer electrolytes with an integrated architecture is a promising pathway toward high-safety, high-performance solid-state lithium-ion batteries.

## 1. Introduction

With the increasing popularity of new energy electric vehicles (EVs) and wearable electronic devices, the development of efficient and safe energy storage devices has become a focus of research [1]. Although liquid lithium-ion batteries (LIBs) have the advantages of high ionic conductivity, good wettability, and compatibility with a variety of anode and cathode electrode materials, they are prone to leakage, are toxic, and are flammable and explosive, which does not meet current safety and environmental protection concepts [2,3]. However, the development of high-safety liquid electrolytes has been limited, and researchers have begun to focus on other directions [4]. Using solid electrolytes to replace liquid organic electrolytes is an effective solution to the above problems [5]. Compared with traditional liquid organic electrolytes, solid electrolytes have advantages such as non-flammability, high thermal stability, high energy density, and environmental friendliness. In the solid-state battery manufacturing process, optimizing the composition of the solid-state electrolyte is critical to improving the overall performance of the battery [6].

Among many types of solid electrolytes, the polyethylene oxide (PEO) in polymers has drawn significant attention due to its excellent flexibility, strong adhesion, and high solubility for lithium salts [7,8]. However, lithium-ion migration in polymers relies on the movement of polymer chains [9]. This primarily occurs in amorphous regions. At room temperature, the molecular structure of PEO exhibits a high degree of order, which slows down the movement of polymer chains, reduces kinetics, and hinders lithium-ion migration, resulting in low ionic conductivity (10^−7^~10^−6^ S·cm^−1^ at 25 °C) [10,11]. In addition, PEO molecules have a low electrochemical window (<3.9 V), which further limits their compatibility with high-voltage electrode materials [12].

To address the inherent defects of PEO, researchers have attempted to modify its ordered structure, reduce its crystallinity, and expand the area of its amorphous regions [13]. The addition of inorganic fillers to PEO is considered the simplest and most effective approach [14]. The incorporation of inorganic fillers can decrease the crystallinity of PEO, enhance the mechanical strength of the electrolyte membrane while improving lithium-ion conductivity, increase the concentration of charge carriers, and provide additional pathways for lithium-ion transport. Li_1+x_Al_x_Ti_2-x_(PO_4_)_3_ (LATP) is an ideal dopant phase for polymer electrolytes due to its wide electrochemical window, high room-temperature ionic conductivity, and excellent thermal stability [15,16]. The introduction of LATP can utilize the ‘scaffold’ effect of ceramic particles to suppress PEO chain relaxation, thereby improving the composite electrolyte’s ionic conductivity and mechanical strength [17].

The incorporation of inorganic fillers into polymer electrolytes has been extensively reported. Yan et al. [18] dispersed nanosized LATP into a PEO polymer matrix via a conventional casting method to obtain composite electrolyte membranes. Their study revealed that while LATP offered only a limited improvement in the ionic conductivity of PEO, it significantly reduced the interfacial resistance between PEO and lithium metal. Nevertheless, degradation of the solid electrolyte/electrode interfacial contact was still observed after prolonged cycling. Luo et al. [19] prepared a porous electrolyte membrane using a polymer–LATP composite, which was then soaked in liquid electrolytes to form a gel electrolyte with excellent interfacial adaptability. Although this approach effectively addressed the contact issue between the electrolyte and electrode, gel electrolytes, as an intermediate state between liquid and solid electrolytes, still fail to fundamentally resolve the interfacial compatibility challenges inherent to solid-state systems. While the introduction of LATP into polymer matrices has been previously reported, studies focusing on optimizing fabrication strategies to construct integrated electrodes for improved interfacial stability remain scarce.

In this work, we prepared a PELT solid electrolyte membrane with an ionic conductivity of 1.2 × 10^−4^ S·cm^−1^ at 60 °C, an electrochemical stability window of 4.9 V, and a tLi+ of 0.46. Furthermore, to address the interfacial contact issues between the electrolyte and electrode observed in previous studies, we innovatively applied a secondary coating method to integrate the PELT solid electrolyte with LFP cathode material, thereby designing and fabricating an electrolyte/electrode-integrated solid-state battery. Compared with conventional layered solid-state batteries, this integrated architecture exhibits significantly improved cycling stability and rate performance, highlighting the crucial role of structural optimization in enhancing the performance of solid-state batteries. We anticipate that this research will provide new insights and approaches for achieving high-performance solid-state batteries.

## 2. Materials and Methods

### 2.1. Preparation of LATP Solid Particles

The LATP was synthesized via a solution-based method. Initially, a 29.2% aqueous ammonia solution was mixed with titanium tetraisopropoxide (TiO_4_C_12_H_28_) under stirring, leading to the formation of a white precipitate. The precipitate was collected by filtration, thoroughly rinsed several times with deionized water to eliminate excess alkali, and subsequently redispersed in fresh deionized water. Upon the gradual addition of 1 mol L^−1^ oxalic acid solution at room temperature, the suspension transformed into a clear solution. Afterwards, ammonium dihydrogen phosphate ((NH_4_)_2_HPO_4_), lithium nitrate (LiNO_3_), and aluminum nitrate nonahydrate (Al(NO_3_)_3_·9H_2_O) were successively introduced. The mixture was then maintained at 80 °C with continuous stirring for 8 h until the white precipitate was completely generated. The obtained material was dried to remove residual moisture, and finally calcined at 700 °C for 12 h, yielding pure white LATP particles.

### 2.2. Preparation of PELT Solid Composite Electrolyte

**Figure 1:**
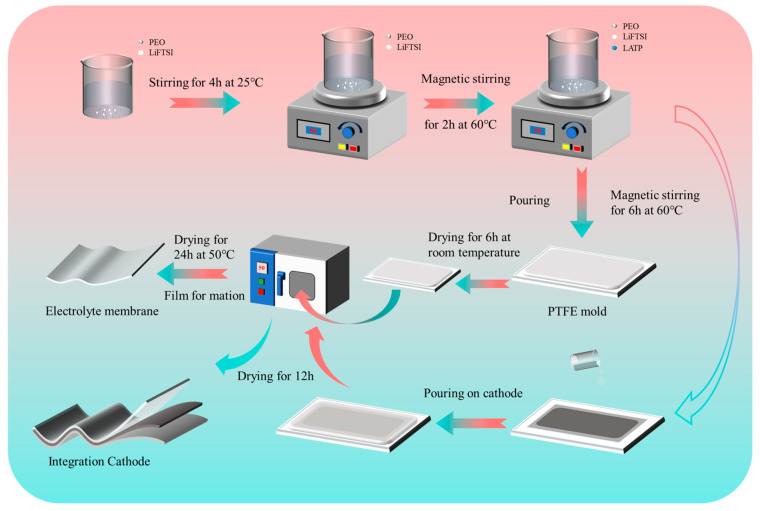
Schematic diagram of the preparation process for solid-state composite electrolyte membranes and integrated electrode/electrolyte structures.

The SCE membranes were fabricated via a solution casting technique. In the first step, 1.0 g of high-molecular-weight PEO (Mw = 600,000) and a certain proportion of lithium salt (LiTFSI) (mass ratio of 1:3 against the mixing polymer) were dispersed in anhydrous acetonitrile under continuous magnetic stirring at 25 °C for 4 h, followed by an additional stirring process at 60 °C for 2 h to ensure sufficient dissolution and coordination between the polymer chains and lithium salt. Subsequently, 0.15 g of LATP ceramic filler was incorporated into the obtained PEO–LiTFSI (PEL) polymer solution, and the mixture was further stirred for 6 h to achieve a homogeneous and stable PELT precursor solution. The as-prepared slurry was carefully cast into a polytetrafluoroethylene (PTFE) mold and left to stand at ambient temperature for 6 h, which allowed for the slow volatilization of the solvent and effectively minimized bubble formation within the membrane. Afterwards, the solidified precursor was transferred into a vacuum oven and dried at 50 °C for 24 h to completely remove the residual solvent, finally yielding the dense and flexible PELT electrolyte membrane. For comparison, PEL membranes containing only PEO and LiTFSI were fabricated using the same procedure without the addition of LATP. All the prepared electrolyte films were subsequently transferred into an argon-filled glovebox, where they were punched into disks with a diameter of 19 mm for subsequent electrochemical characterization and battery assembly (Figure 1).

### 2.3. Preparation of In Situ Integrated Cathode

The cathode was prepared by mixing LiFePO_4_, Ketjen Black conductive carbon, and a polyvinylidene fluoride (PVDF) binder in a mass ratio of 8:1:1. The mixture was thoroughly dispersed in an appropriate solvent and subjected to continuous stirring for 12 h to ensure intimate contact among the active material, conductive additive, and polymer binder, thereby forming a uniform and stable electrode slurry. The well-prepared slurry was subsequently cast onto aluminum foil current collectors using a coating machine, followed by drying in a vacuum oven at 80 °C for 24 h to remove the residual solvent and to obtain a dense LFP electrode film. To construct an integrated electrode–electrolyte structure, a thin layer of PELT precursor solution was uniformly coated onto the surface of the dried LFP cathode. The coated electrodes were left undisturbed at room temperature for 6 h, allowing the precursor solution to gradually infiltrate into the electrode pores and establish sufficient interfacial contact. Thereafter, the electrodes were transferred to a vacuum oven and dried at 50 °C for 12 h, ensuring complete solvent removal and the formation of a compact composite interface. Through this process, an integrated SSLB was successfully obtained (Figure 1). Finally, the assembled electrodes were moved into an argon-filled glove box to prevent moisture or oxygen contamination, and circular electrode disks with a diameter of 16 mm were carefully punched out using a precision cutting machine for subsequent electrochemical measurements and performance evaluations.

### 2.4. Materials Characterization

The prepared SCE membrane was tested for crystallinity using X-ray diffraction (XRD, Rigaku Ultima IV, Tokyo, Japan) with a test angle range of 10~60° and a scanning speed of 10°/min. The morphology and microstructure of the solid composite electrolyte were observed using a field emission scanning electron microscope (SEM, ZEISS Sigma 360, Oberkochen, Germany), and elemental analysis was performed using energy-dispersive spectroscopy (EDS). The thermal stability of the composite solid electrolyte was measured using differential scanning calorimetry (DSC, DSC214 Polyma, Selb, Germany) from −80 °C to 100 °C under a nitrogen atmosphere. A UTM4103 tensile tester was used to test the mechanical properties of the solid electrolyte membrane. The chemical composition of LFP after one cycle was analyzed using X-ray photoelectron spectroscopy (XPS, Thermo Fisher Scientific K-Alpha, Waltham, MA, USA).

### 2.5. Electrochemical Measurements

The assembly of stainless steel (SS)//electrolyte membrane//SS symmetric batteries and the calculations of ionic conductivity of the SCE was performed using impedance testing. The impedance values of PEL and PELT were measured using an electrochemical workstation (CHI760F) with a frequency range of 1 Hz to 10^6^ Hz and an amplitude voltage of 5 mV. The measured impedance value R was substituted into Equation (1) to calculate the ionic conductivity of the test SCE. In this equation, L denotes the thickness of the SCE membrane and S represents the area of the SS plate [20].

(1) $$\sigma =\frac{L}{RS}$$

Coin batteries were assembled with SS as the working electrode and lithium metal as the counter electrode, and the electrochemical stability window of the SCE was tested using linear sweep voltammetry (LSV). The scan rate was 1 mV·s^−1^ and the test voltage range was 2~6 V.

To assemble lithium–lithium symmetric batteries, test lithium-ion migration numbers, and evaluate ion migration capacity, we applied a constant polarization voltage ΔV (10 mV) to the symmetric batteries, recorded changes in current over time, and measured the initial and steady-state interface impedance of the lithium–lithium symmetric batteries using Equation (2) [19].

(2) $$t_{Li+}=\frac{I_{ss}(\Delta V-I_{0}R_{0})}{I_{0}(\Delta V-I_{ss}R_{ss})}$$

In Equation (2), ΔV denotes the polarization voltage, I_0_ refers to the initial current, Iss stands for the steady-state current, and R_0_ and Rss correspond to the initial and steady-state interface impedances, respectively.

Additionally, the LANHE battery testing system was used to conduct constant current charge–discharge tests to test the stability of the interface between the electrodes and the SCE. Under 60 °C conditions, the cycling and rate performance of lithium-ion batteries with a “sandwich” structure using PELT composite polymer electrolyte membranes and a “monolithic” structure using a new PEO-based composite polymer electrolyte were tested to investigate the impact of different battery structures on battery performance.

## 3. Results and Discussion

**Figure 2:**
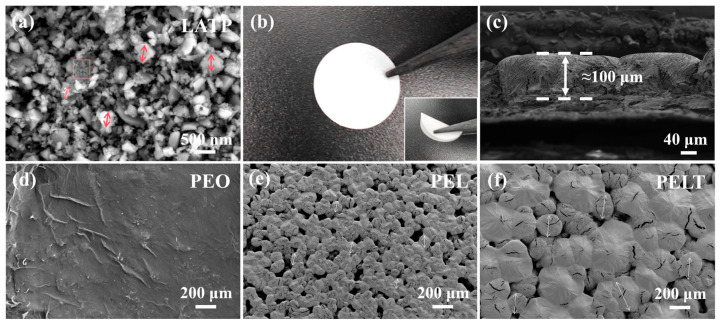
(**a**) SEM image of LATP; (**b**) optical micrograph of PELT, with a bent image in the bottom right corner; (**c**) SEM image of PELT cross-section; (**d**) SEM image of pure PEO; (**e**) SEM image of PEL; (**f**) SEM image of PELT.

SEM was used to observe the microscopic morphology of the composite. Figure 2a presents the microstructure of LATP, where most intact particles exhibit an irregular block-like morphology with sizes ranging from 200~500 nm. In addition, smaller particles with dimensions of approximately 20~100 nm can also be observed in the selected region. The introduction of nano-sized inorganic fillers can provide more ionic transport channels for the polymer, thereby improving the ionic conductivity of PEO-based electrolytes [21]. Figure 2b shows an optical image of the PELT solid-state composite electrolyte. As can be seen from the figure, the surface of the electrolyte membrane is smooth and flat, and the membrane exhibits good flexibility upon bending after high-temperature treatment. The flexible electrolyte membrane in contact with the electrode helps reduce interfacial impedance. Figure 2c shows the cross-sectional image of the PELT membrane following the incorporation of LATP, from which the thickness of the PELT membrane is approximately 100 μm. The surface of the pure PEO polymer membrane is smooth, forming a compact and integrated structure without noticeable aggregation or voids (Figure 2d). Figure 2e also includes the image of the PEO polymer electrolyte with LiTFSI added (PEL). As observed, the PEO polymer encapsulates most of the lithium salt, forming polymer microspheres with more voids formed on their surfaces. This formation of voids mainly originates from the solution casting process, where the relatively rapid evaporation of acetonitrile leads to incomplete packing of the PEO chains and partial phase separation between PEO and LiTFSI, thereby resulting in uniformly distributed voids. After the addition of LiTFSI, the microscopic morphology of the PEO polymer electrolyte thus exhibits a relatively continuous and intact network structure.

Surface observation of the PELT electrolyte membrane (Figure 2f) reveals a flat surface with uniform thickness. The introduced LATP fillers are encapsulated by the polymer matrix as well, contributing to the formation of larger polymer microspheres. PEL microspheres exhibit diameters of 70~100 μm, while PELT microspheres show larger sizes of 200~300 μm. Normally, the introduction of LATP would increase the voids in the electrolyte membrane. However, in this study, the effect of the processing conditions led to an increase in the size of the polymer microspheres, confirming that the LATP particles were well encapsulated by the polymer. This not only promotes the growth of the microspheres but also partially fills the voids on the membrane surface, thereby enhancing the interfacial contact between the electrolyte membrane and the electrode and facilitating ion transport. [22]. Additionally, no LATP particles are detected on the membrane surface, indicating that most inorganic fillers are embedded inside the membrane, encapsulated by PEO and LiTFSI.

**Figure 3:**
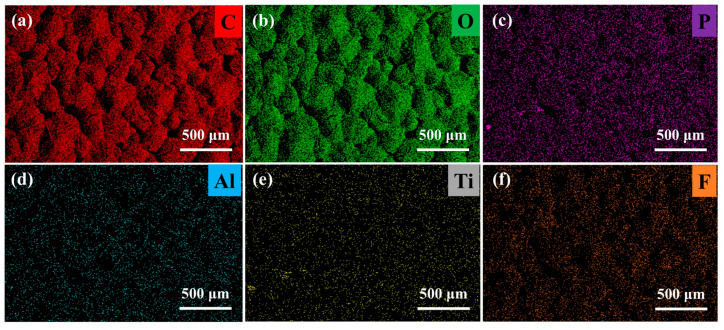
EDS elemental mappings of (**a**) C; (**b**) O; (**c**) P; (**d**) Al; (**e**) Ti, and (**f**) F elements in the electrolyte membrane.

To further analyze the distribution of LATP in the PEO electrolyte, EDS analysis was performed on the PELT electrolyte membrane (Figure 3). As shown, LATP is uniformly dispersed in the polymer matrix with no obvious agglomeration. Such uniform distribution can enhance the overall mechanical strength of the electrolyte membrane. Moreover, it can induce uniform lithium deposition during cycling, thereby reducing the growth of lithium dendrites [23].

**Figure 4:**
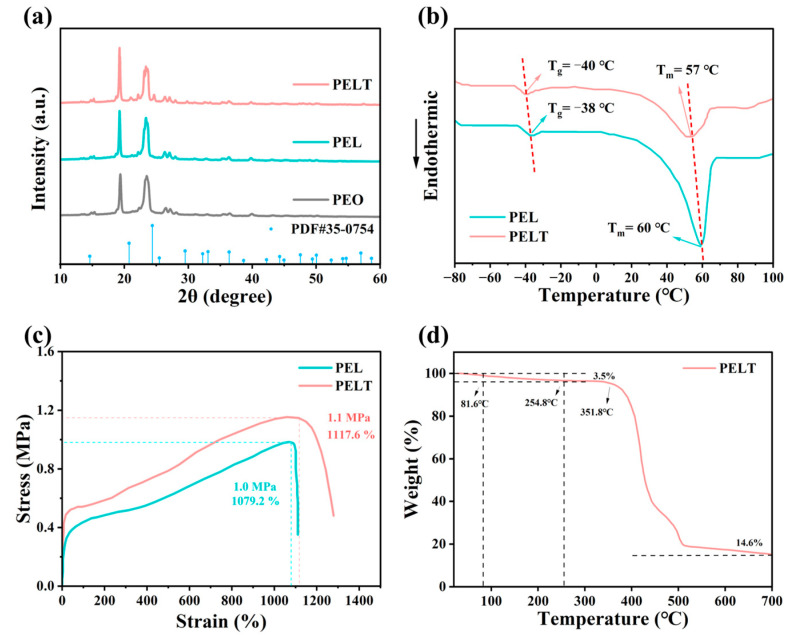
(**a**) XRD patterns of PEO-based electrolytes; (**b**) DSC curves of PEL and PELT; (**c**) stress–strain curves of PEL and PELT; (**d**) TGA curve of PELT.

XRD was used to characterize the PEO-based electrolytes (Figure 4a). Since LiTFSI exists in an amorphous form, no characteristic peaks of LiTFSI are observed in the XRD patterns [24]. PEL is obtained by adding LiTFSI to PEO. By comparing the XRD patterns, it is seen that the intensity of diffraction peaks in PEL is lower than that in PEO. This is because Li^+^ coordinates with the ether oxygen groups in PEO molecular chains, disrupting the ordered arrangement of polymer molecular chains and reducing the crystalline regions of the PEO matrix, thus leading to the weakened intensity of diffraction peaks [25]. Further observation of the XRD pattern of the PELT electrolyte after adding LATP shows that with the addition of LATP, the diffraction peaks of the inorganic filler (JCPDS 35-0754) gradually increase in intensity, while the intensity of PEO’s characteristic diffraction peaks further decreases. This indicates that the introduction of LATP further suppresses the crystallinity of PEO. The higher the proportion of amorphous regions, the more favorable it is for the migration of lithium ions in the polymer, which improves the ionic conductivity of the electrolyte [26].

DSC was used to investigate the phase transition behavior of the electrolyte membranes. As illustrated in Figure 4b, the PEL electrolyte exhibits a glass transition temperature (Tg) of −38 °C and a melting temperature (Tm) of 60 °C, whereas the Tg and Tm of PELT are −40 °C and 57 °C, respectively, both lower than the corresponding values of PEL. This further indicates that the incorporation of LATP can suppress the crystallinity of the PEO-based electrolyte. The decrease in Tm implies a reduction in the crystallinity of the electrolyte membrane, while the lower Tg reflects an increase in the amorphous regions, which is more conducive to the migration of lithium ions within the polymer matrix [27].

Figure 4c presents the stress–strain curves of PEL and PELT electrolyte membranes. As can be seen from the figure, the maximum stress and strain of the PEL electrolyte membrane are 1.0 MPa and 1079.2%, respectively, while those of PELT are 1.1 MPa and 1117.6%, respectively. This indicates that the introduction of LATP can enhance the overall mechanical properties of the electrolyte membrane; the membrane thus gains better puncture resistance, which can effectively block the growth and piercing of lithium dendrites during the cycling process [28].

From the TGA curve of PELT, a slight weight loss is observed from room temperature to 81.6 °C, which can be attributed to the release of adsorbed moisture arising from the slight hygroscopicity of LiTFSI. A further weight decrease is detected up to 254.8 °C, corresponding to the evaporation of residual acetonitrile and the partial decomposition of low-molecular-weight PEO. Although the boiling point of acetonitrile is around 81 °C, its retention within the polymer matrix or pores often delays its complete volatilization to higher temperatures [29]. Upon heating to 351.8 °C, pronounced weight loss occurs, which is primarily associated with the thermal degradation of the PEO main chain. Ultimately, a residual weight of 14.6% remains, mainly originating from LATP and other inorganic constituents in the system (Figure 4d).

**Figure 5:**
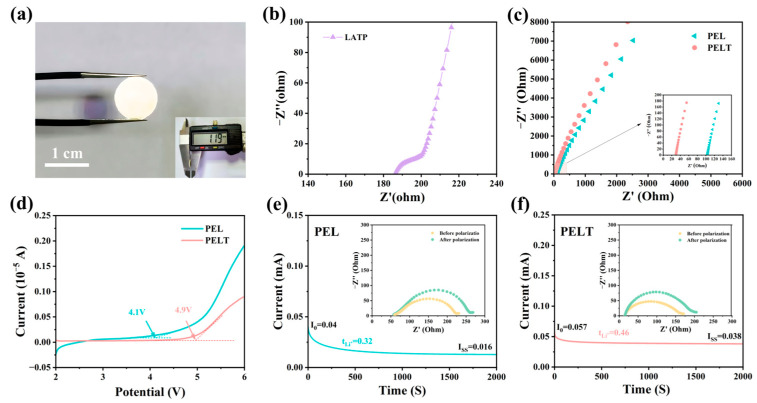
(**a**) Optical images of pure LATP; (**b**) EIS curve of pure LATP; (**c**) EIS curves of PEL and PELT at 60 °C; (**d**) LSV curves of PEL and PELT; (**e**) galvanostatic polarization curve of the battery with PEL electrolytes, and EIS curves before and after polarization; (**f**) galvanostatic polarization curve of the battery with PELT electrolytes, and EIS curves before and after polarization.

By measuring the diameter of pelletized pure LATP electrolytes and calculating from the EIS spectra, we determined that the ionic conductivity of LATP at 60 °C is 8.6 × 10^−4^ S cm^−1^ (Figure 5a,b). The effect of LATP on the ionic conductivity of the solid-state electrolyte was investigated using EIS. As can be seen from Figure 5c, the impedance values of PEL and PELT at 60 °C are 104.3 Ω and 29.8 Ω, respectively. It is evident that the impedance of the electrolyte membrane decreases with the introduction of LATP. By substituting these impedance values into Formula (1), the calculated ionic conductivities of PELT and PEL are 1.2 × 10^−4^ S·cm^−1^ and 4.5 × 10^−5^ S·cm^−1^, respectively. Moreover, as discussed in the manuscript, the introduction of LATP as an inorganic filler not only provides additional ion-conduction pathways, but also alters the crystallinity of the PEO matrix, increasing the proportion of amorphous regions and thereby enhancing the segmental mobility of PEO chains. This observation is consistent with our original statement regarding “enhanced segmental motion.” Taken together, the improvement in ionic conductivity of the composite electrolyte arises from both the intrinsically high conductivity of LATP and its ability to promote PEO chain mobility, demonstrating a clear synergistic effect.

Batteries with a SS//SCE//Li structure were assembled, and LSV tests were conducted within a potential range of 2 to 6 V to investigate the electrochemical window of the solid electrolytes. As shown in Figure 5d, for the PEL electrolyte, the oxidation current increases sharply when the voltage reaches 4.1 V, which is caused by oxidation reactions. LATP inorganic fillers possess a wide electrochemical window; incorporating LATP into the PEO matrix is conducive to increasing the oxidative decomposition voltage of the battery. Compared with the PEL electrolyte, the composite electrolyte PELT exhibits an electrochemical window as high as 4.9 V. The LSV test results further demonstrate that the composite electrolyte can enhance the cycling safety and stability of the battery.

Li//Li symmetric batteries were assembled using the electrolyte membranes, and galvanostatic polarization was employed to investigate the tLi+ of the electrolyte membranes. Figure 5e,f display the galvanostatic polarization curves of cells with PEL (c) and PELT (d) electrolytes, with the insets showing EIS images before and after polarization. Calculated via Formula (2), the tLi+ values of PELT and PEL are 0.46 and 0.32, respectively. The tLi+ of PELT is higher than that of PEL, which can be attributed to multiple synergistic effects. First, LATP is an intrinsically fast Li^+^ conductor, and when dispersed in the PEO matrix, it can form interfacial transport channels or partially percolated networks that provide parallel pathways for Li+ migration. Second, the surface of LATP contains Lewis acidic sites such as Ti^4+^ and Al^3+^, which are capable of interacting with TFSI^−^ anions and thereby suppressing their mobility [30]. When anion diffusion is hindered while Li^+^ mobility is maintained or even improved, the overall tLi+ of PELT increases. In addition, the incorporation of LATP disrupts the crystallinity of PEO, promoting a more amorphous polymer phase that enhances segmental motion, facilitates the dissociation of LiTFSI, and further improves Li^+^ transport within the polymer matrix. Taken together, these factors synergistically contribute to the higher tLi+ observed in PELT.

The performance comparison of the PELT in this work with polymer-based solid-state electrolytes reported in other studies is shown in Table 1, demonstrating the performance advantages of PELT.

**Figure 6:**
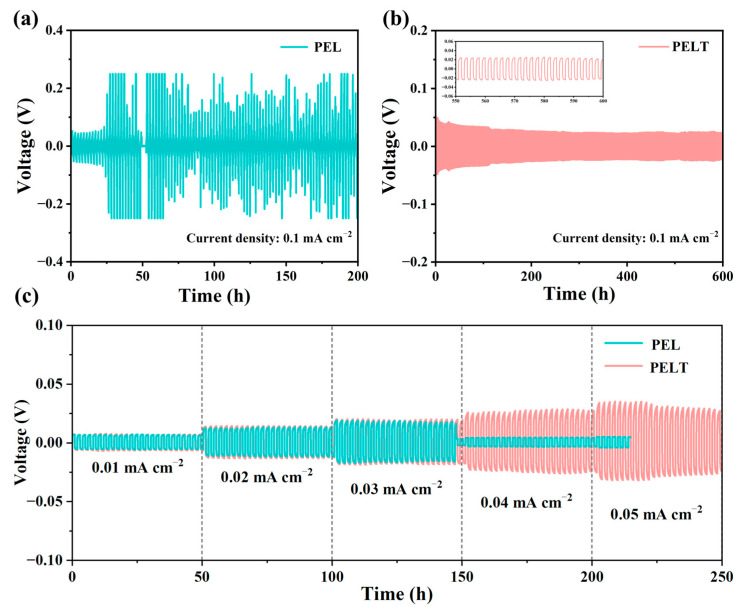
Galvanostatic cycling curves of electrolyte membranes at a constant current density of 0.1 mA·cm^−2^: (**a**) PEL; (**b**) PELT; (**c**) galvanostatic cycling curves of electrolyte membranes under varying current densities.

To systematically investigate the interfacial stability of the electrolyte membranes against lithium metal anodes, Li//Li symmetric batteries were assembled and subjected to long-term galvanostatic cycling tests under both constant and gradually varying current densities. This evaluation is critical, as the formation of lithium dendrites and the resulting short-circuiting remain the major challenges for polymer-based solid electrolytes. Figure 6a,b present the voltage–time profiles of symmetric batteries employing PEL and PELT electrolytes at a constant current density of 0.1 mA·cm^−2^. The PEL-based battery showed a rapid increase in polarization, with the overpotential rising sharply after only 25 h of cycling. This is primarily attributed to the softness of PEL, which can buffer minor interfacial fluctuations, but it is prone to local roughening, microvoid formation, and dendrite growth, which amplifies defects and exacerbates polarization. In addition, its low tLi+ can lead to local concentration gradients at the interface, further increasing the polarization voltage. For the PELT-based battery, the voltage response remained remarkably stable, with no significant fluctuations even after 600 h of continuous cycling. Such stable performance clearly demonstrates the capability of the PELT membrane to effectively suppress lithium dendrite propagation and to maintain a robust electrode–electrolyte interface [36]. This is because the high mechanical modulus of PELT helps resist dendrite penetration; however, dendrite suppression also strongly depends on uniform Li^+^ transport at the interface. The incorporation of LATP not only enhances the mechanical strength but also increases tLi+ and homogenizes Li^+^ flux, reducing local current density peaks. Therefore, the suppression of lithium dendrites arises from the synergistic effect of mechanical reinforcement and improved ionic transport.

To further assess performance under more demanding conditions, stepwise current density tests were conducted (Figure 6c), where the applied current was gradually increased from 0.01 to 0.05 mA·cm^−2^. For the PEL electrolyte, the cycling life was severely limited: after 147 h of operation, once the current density reached 0.04 mA·cm^−2^, the voltage profile collapsed abruptly, indicating internal short-circuiting. This failure can be attributed to lithium dendrites penetrating the relatively weak polymer electrolyte layer, thereby destroying the integrity of the symmetric battery [37]. In contrast, the PELT-based electrolyte delivered markedly superior stability, maintaining smooth voltage profiles for up to 250 h of cycling and showing no signs of short-circuiting even at the maximum tested current density of 0.05 mA·cm^−2^. In the previous impedance tests, PELT exhibited lower resistance owing to the enhanced ionic conductivity induced by LATP. However, under current densities of 0.01~0.03 mA cm^−2^, PELT displayed a slightly higher polarization potential. This could be attributed to the partial reduction in the electronic insulation of PEO upon LATP incorporation, leading to undesirable electronic transport. In addition, side reactions between LATP and lithium metal may form a high-impedance interfacial layer, further contributing to the observed polarization.

Overall, these results provide compelling evidence that the incorporation of nano-sized LATP fillers into the PEO–LiTFSI matrix not only enhances ionic conductivity but also reinforces the mechanical strength and dendrite-blocking ability of the electrolyte.

**Figure 7:**
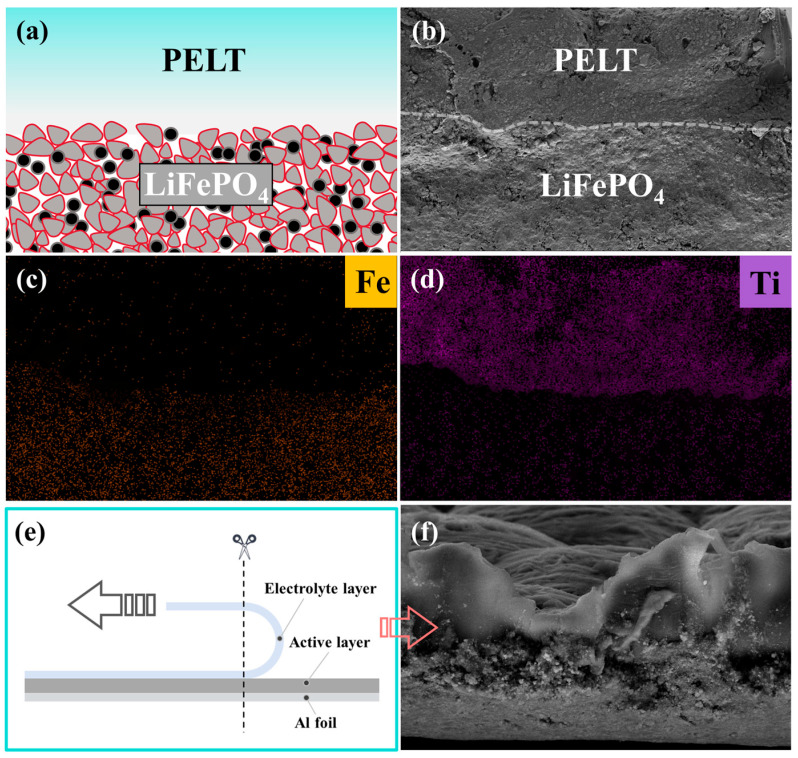
(**a**) Schematic cross-section of PELT/LFP integrated electrode; (**b**) cross-sectional SEM image of the PELT/LFP integrated electrode; (**c**,**d**) elemental mapping distributions of Fe and Ti, respectively, demonstrating the interfacial element diffusion and structural integration; (**e**) schematic diagram of the stripping experiment; (**f**) SEM image of the cross-section of the PELT/LFP integrated electrode after the stripping experiment.

To gain deeper insight into the interfacial architecture of the integrated electrode (Figure 7a), SEM in combination with elemental mapping analysis was employed. These techniques were used to evaluate the degree of interfacial adhesion, which plays a decisive role in determining the efficiency of lithium-ion migration across the electrode–electrolyte boundary. As shown in Figure 7b, the cross-sectional SEM image of the PELT/LFP integrated electrode clearly demonstrates the stratified morphology of the composite structure. Meanwhile, the corresponding elemental mapping results for Fe and Ti reveal their uniform spatial distributions, further confirming that the PELT electrolyte layer and the LFP electrode are well integrated at the microscopic level (Figure 7c,d). Importantly, no visible interfacial gaps, cracks, or delamination phenomena were observed, indicating that the electrolyte and electrode establish intimate physical contact and stable interfacial bonding during the fabrication process.

In order to further validate the robustness of this interfacial adhesion, a peeling test was performed, followed by cross-sectional cutting of the integrated electrode (Figure 7e). The SEM cross-sectional micrograph obtained after the peeling process shows that the PELT electrolyte layer remains firmly attached to the LFP electrode, with the two layers peeling off as a single unit rather than separating independently (Figure 7f). This observation provides direct experimental evidence for the strong interfacial cohesion between the polymer–ceramic composite electrolyte and the cathode material. Such tightly bonded architecture not only ensures mechanical durability during repeated electrochemical cycling but also maximizes the effective contact area between the two components. As a result, the interfacial resistance is markedly reduced, and the transport kinetics of lithium ions across the electrode–electrolyte interface are significantly accelerated.

Collectively, these results highlight that the integrated electrode design achieves excellent interfacial compatibility and structural stability. This optimized interface plays a critical role in promoting efficient ion transfer, mitigating interfacial polarization, and ultimately contributing to the superior electrochemical performance of the assembled solid-state lithium batteries.

**Figure 8:**
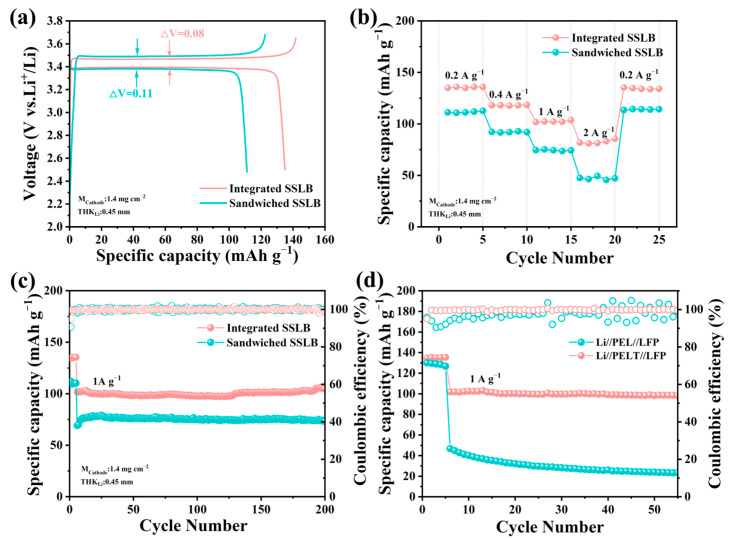
Electrochemical performance of SSLBs: (**a**) polarization voltage curves of sandwiched-type and integrated-type batteries; (**b**) rate performance curves of sandwiched-type and integrated-type batteries; (**c**) cycling performance curves of sandwiched-type and integrated batteries; and (**d**) cycling performance curves of PEL and PELT batteries.

To investigate the electrochemical performance of SSLB, two types of batteries were fabricated: sandwiched-structured solid-state lithium-ion batteries (Sandwiched SSLBs, where LFP cathodes and PELT electrolytes were laminated separately) and Integrated SSLBs. As shown in Figure 8a, the initial discharge capacity of the Integrated SSLB reaches 134.8 mAh·g^−1^, significantly higher than that of the Sandwiched SSLB (111.1 mAh·g^−1^), and the polarization voltage (ΔV) of the Integrated SSLB is only 0.08 V, much lower than the 0.11 V of the Sandwiched SSLB, indicating that the integrated architecture optimizes the solid–solid interface contact, enabling more efficient transport of both lithium ions and electrons across the electrode–electrolyte boundary [38].

For rate performance (Figure 8b), at a low current density of 0.2 A·g^−1^, the Integrated SSLB retains a discharge capacity of 135 mAh·g^−1^, while the Sandwiched SSLB exhibits a lower capacity of 111.2 mAh·g^−1^, and when the current density increases to 2 A·g^−1^, the Integrated SSLB maintains 85.4 mAh·g^−1^ (corresponding to a capacity retention of 63% relative to 0.2 A·g^−1^), whereas the Sandwiched SSLB only retains 47.4 mAh·g^−1^ (with a capacity retention of 43%), with the superior rate capability of the Integrated SSLB stemming from its monolithic structure that minimizes interfacial impedance and facilitates rapid lithium ion migration even under high-current conditions.

Cycling tests conducted at 1 A·g^−1^ (Figure 8c) further highlight the advantage of the integrated architecture: after 200 cycles, the Integrated SSLB retains a discharge capacity of 104.8 mAh·g^−1^ (with a capacity retention of 74%), demonstrating excellent cycling stability, while in contrast, the Sandwiched SSLB only retains 73.4 mAh·g^−1^ (a capacity retention of 66%) after the same number of cycles. With identical electrodes, the performance gap arises from the electrolyte–electrode interface: for the same PEO-based electrolyte, the integrated structure enhances contact, reduces impedance, and significantly boosts battery performance. After confirming the superiority of the sandwich structure, the electrochemical performance of the integrated PEL asymmetric battery was further investigated. In terms of cycling performance, the PELT-based battery maintained a higher capacity retention than the PEL-based battery after 50 cycles, further validating its advantages in electrochemical performance (Figure 8d). Overall, owing to its superiority in both rate capability and cycling stability, the PELT system highlights the significant optimization effect of LATP modification on PEO-based solid electrolytes, making it a more promising electrolyte candidate compared to PEL.

**Figure 9:**
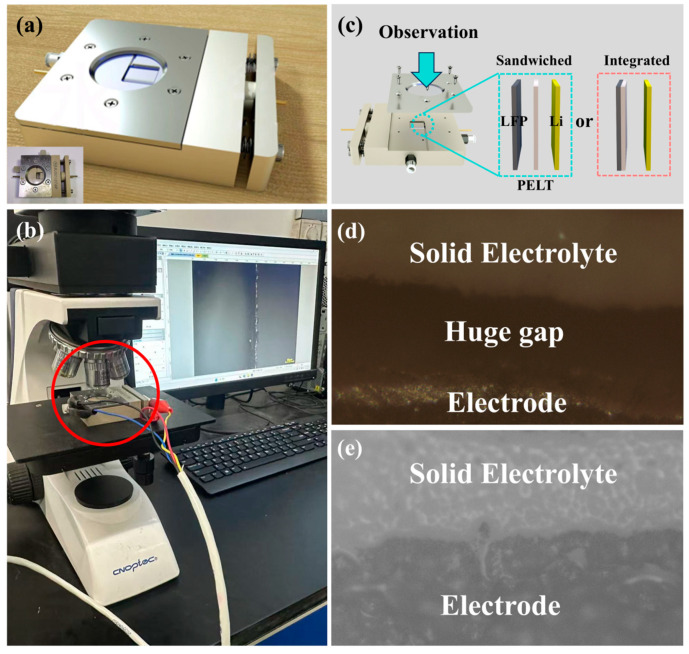
In situ optical testing of lithium battery: (**a**) In situ battery; (**b**) in situ system assembly demonstration; (**c**) schematic diagram of in situ battery electrode assembly; (**d**) optical photograph of the electrolyte/cathode interface in a sandwich structure battery after 50 cycles; and (**e**) optical photograph of the electrolyte/cathode interface in a sandwich structure battery after 50 cycles.

To further verify the superiority of the integrated electrolyte/electrode structure, we employed a customized in situ battery device to observe the interfacial behavior of both conventional Sandwiched-type batteries and the newly developed integrated batteries. The operating principle of the in situ battery is identical to that of conventional batteries, with the distinction that it allows for longitudinal observation of the interfacial evolution among different components (Figure 9a). By coupling the in situ battery with a charge–discharge system and a metallographic microscope, the interfacial changes during cycling can be directly visualized (Figure 9b). The assembly process was consistent with that of other batteries. For conventional sandwich batteries, the components were sequentially stacked in the order of LFP, PELT, and Li. In contrast, the integrated batteries were assembled by directly pairing the integrated electrode with Li, thereby simplifying the assembly procedure. This streamlined process also highlights the particular advantages of integrated batteries in improving manufacturing efficiency and reducing process-related errors (Figure 9c).

Both sandwich and integrated batteries were cycled at a current density of 0.5 mA·cm^−2^ for 50 cycles. A comparison of their optical images revealed that the sandwich batteries exhibited significant voids, which can be attributed to the inherent limitations of the traditional stacking approach—namely, the difficulty of achieving complete integration between the solid electrolyte and the electrode after cycling. In contrast, the integrated batteries maintained a tightly bonded electrolyte/electrode interface even after prolonged cycling (Figure 9d,e). This interfacial evolution is in good agreement with the electrochemical results discussed earlier: the insufficient interfacial contact in sandwich-type batteries leads to uneven ion transport and aggravated polarization, thereby accounting for their inferior rate capability and cycling stability. Conversely, the intimate and robust interface in integrated batteries effectively minimizes interfacial resistance and ensures uniform ion flux, which underpins their superior electrochemical performance.

## 4. Conclusions

In conclusion, this work successfully developed a PELT composite solid electrolyte together with an integrated electrode design, both of which are highly promising for the fabrication of stable and safe solid-state lithium-ion batteries. The incorporation of nano-sized LATP ceramic fillers into the PEO polymer matrix not only suppressed the intrinsic crystallinity of PEO but also enhanced the flexibility and mechanical robustness of the electrolyte membrane. More importantly, the introduction of LATP provided abundant lithium-ion transport channels, thereby significantly improving ionic mobility within the polymer framework. As a result, the prepared PELT electrolyte exhibited a wide electrochemical stability window of up to 4.9 V and a high ionic conductivity of 1.2 × 10^−4^ S·cm^−1^ at 60 °C, both of which serve as key prerequisites for achieving long-term and reliable battery performance.

Beyond electrolyte optimization, this study also proposed a rational design strategy for integrated electrodes. Specifically, by adopting an in situ casting approach to coat the PELT precursor directly onto the LFP cathode surface, intimate interfacial contact was established between the electrode and the electrolyte. This configuration effectively minimized interfacial impedance, suppressed potential side reactions at the interface, and thereby enhanced the overall electrochemical stability of the assembled solid-state lithium battery. The resulting SSLB demonstrated improved cycling durability and operational safety, highlighting the dual advantages of both electrolyte modification and electrode integration.

Overall, the findings of this study provide not only a straightforward and scalable route for the preparation of polymer–ceramic composite electrolytes but also a practical strategy for electrode–electrolyte integration. Such advancements hold significant potential for accelerating the large-scale application of SSLBs, particularly in areas requiring high safety and energy density, such as electric vehicles, grid-scale energy storage, and next-generation portable electronic devices.

## Figures and Tables

**Table 1:** Comparison of the electrochemical performance of PELT with other reported polymer electrolytes.

Polymer Electrolyte	Ionic Conductivity (S·cm^−1^)	Stable Voltage Window (V)	Li Transference Number (tLi+)	Ref.
PVDF-HFP	1.73 × 10^−5^	4.1	0.27	[31]
PAN-GO	1.1 × 10^−4^	5.0	0.40	[32]
PVCA-LiDFOB	9.82 × 10^−5^	4.5	0.57	[33]
P(EO-POSS)	9.5 × 10^−5^	4.1	0.25	[34]
PCPU	1.12 × 10^−4^	4.5	0.45	[35]
PELT	1.2 × 10^−4^	4.9	0.46	This work

## Data Availability

The original contributions presented in this study are included in the article. Further inquiries can be directed to the corresponding authors.
